# Vaccination Strategies at a COVID-19 Mass Vaccination Site

**DOI:** 10.34172/ijhpm.2022.6881

**Published:** 2022-02-26

**Authors:** Sheng-Huang Hsiao, Sheng-Jean Huang, Chiao-Yu Huang

**Affiliations:** ^1^Department of Neurosurgery, Taipei City Hospital, Taipei, Taiwan.; ^2^Department of surgery, Medical College, National Taiwan University, Taipei, Taiwan.; ^3^Department of Family Medicine, Taipei City Hospital RenAi Branch, Taipei, Taiwan.; ^4^Department of Education and Research, Taipei City Hospital, Taipei, Taiwan.; ^5^Department of Oral Hygiene and Healthcare, Cardinal Tien College of Health and Management, New Taipei City, Taiwan.

## Dear Editor,

 Planning and implementing mass vaccination is important and challenging in regard to the control of Coronavirus disease 2019 (COVID-19).^1-4^ In addition to traditional points of dispensing, such as clinics or hospitals, high-throughput large venues, such as arenas, have been used to rapidly immunize a large number of people in many countries.^5-7^ Since the COVID-19 outbreak that occurred in May 2021, Taiwan has been racing to vaccinate as many citizens as possible. The Taipei Expo Park’s Expo Dome, which is an exhibition hall that was once used for the 2010 Taipei International Flora Exposition, was transformed into one of the first mass vaccination sites in Taiwan starting in July 2021.

 The Taipei Expo Park, which is positioned adjacent to a transport hub in Taipei, is a barrier-free environment that is easily accessible to the public.^8^ The Expo Dome measures 6030 square meters (106 meters by 60 meters) and was thus able to accommodate 18 service blocks and their stations, a command post and areas for supply storage (Figure, left). In each service block, 84 chairs (3 by 28) were placed one meter apart to maintain social distancing.^9^ A vaccination team with one physician, two nurses and nonmedical staff was placed in charge of one or two service blocks. Physicians and nurses were responsible for prevaccination evaluations, shot dispensing and potential treatment for acute adverse events after vaccination. Nonmedical staff were responsible for triage, registration and channeling vaccine recipients.

**Figure:**
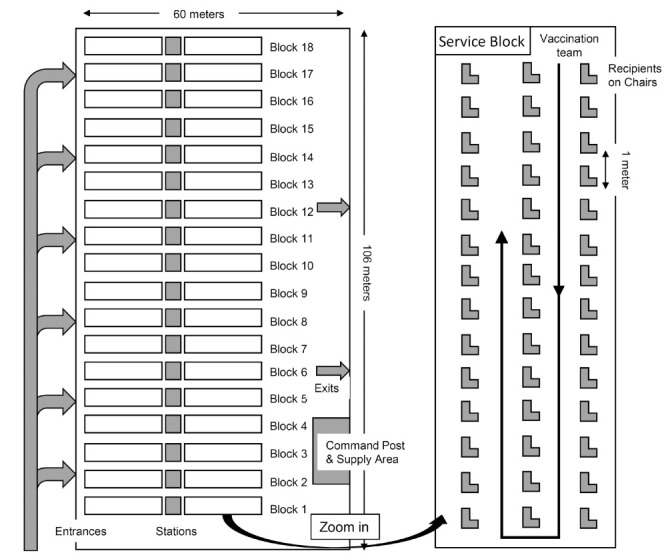
The 2D Layout of the Expo Dome Mass Vaccination Site (Left) and a Schematic Diagram of the Vaccine Administration Method, Namely, the Umi-machi Method, Used Within in a Service Block (Right).

 Mass vaccination in the Expo Dome adopted an innovative vaccine administration method, namely, the Umi-machi method, which originated in a town in Japan.^10^ In this method, the vaccine recipient remains seated in the same chair in a service block during the whole inoculation process and observation period after vaccination (Figure, right). The vaccination team, wearing personal protection equipment, moves from one person to another for registration, individualized evaluation and shot dispensing. The idea for this method, which was derived from vehicle assembly lines, limits the movement of vaccine recipients while maintaining efficiency. For each recipient, the process takes an average of 2 minutes for registration, 1 minute for shot dispensing and 15 to 30 minutes for observation. The total median length of stay for each recipient in the Expo Dome was 25 minutes. On average, a vaccination team could deliver 80 to 150 vaccinations in one hour. The comparison between the Umi-machi method and the traditional vaccine administration method is shown in Table.

**Table:** The Comparison Between the Umi-machi Method and the Traditional Vaccine Administration Method

	**Umi-machi Method**	**Traditional Vaccine Administration Method**
Characteristics	Vaccine recipients remain seated in the same chair and the vaccination team move from one person to another during the whole process	Vaccine recipients line-up and move around for registration, inoculation and observation after vaccination
Required workforce	Larger workforce, collaboration among healthcare personnel and nonmedical staff	Mainly healthcare personnel
The speed of vaccination	Faster, 80 to 150 vaccinations in 1 hour	Slower, depending on the traffic flow planning
Contact among participants	Low contact, with social distancing	Close contact if crowd congestion or in a small space
Acceptance of people	Good acceptance, especially among the elderly and those with disabilities	Widely accepted and used in routine practice

 The Taipei government incorporated mass vaccination at the Expo Dome as part of the city’s inoculation program. Citizens could make an appointment on a government-funded online reservation platform to receive their COVID-19 vaccinations at either the Expo Dome or traditional points of dispensing. The Expo Dome operated 3 to 8 hours a day, with 2 to 18 service blocks open every working day, depending on the number of vaccine recipients. From July 14 to September 25 in 2021, the Expo Dome served a total of 124 301 vaccine recipients (ranging from 251 to 8041 recipients per day) in 45 working days.

 The main advantages of the mass vaccination strategy used in the Expo Dome include (1) large throughput with good crowd control, (2) faster vaccination under lower disease transmission risk than traditional walk-in clinics, and (3) person-centered service and comfort for recipients, especially elderly individuals and those with disabilities. However, the operation of such a program not only requires coordination and partnership of the government with the health care system and different stakeholders, such as logistics and social media, but it also needs a large amount of space and a suitable infrastructure. The experience yielded from this mass vaccination program is critical for pandemic control and can be used as a reference for countries seeking to achieve population-wide vaccination.

## Acknowledgements

 The authors thank all staff members in Taipei City Hospital that involved in the work at the Taipei Expo Dome.

## Ethical issues

 Not applicable.

## Competing interests

 Authors declare that they have no competing interests.

## Authors’ contributions

 SHH, SJH, and CYH all contributed to the article writing.
